# CM-UNetv2: An Enhanced Semantic Segmentation Model for Precise PCB Defect Detection and Boundary Restoration

**DOI:** 10.3390/s25164919

**Published:** 2025-08-09

**Authors:** Qiyang Guo, Yajun Chen, Yirui Zhu, Dongle Chen

**Affiliations:** Department of Information Science, Xi’an University of Technology, Xi’an 710048, China; 2230821075@stu.xaut.edu.cn (Q.G.); 2240820017@stu.xaut.edu.cn (Y.Z.); 2240821096@stu.xaut.edu.cn (D.C.)

**Keywords:** PCB, semantic segmentation, multi-scale feature extraction, neural networks

## Abstract

PCBs play a critical role in electronic manufacturing, and accurate defect detection is essential for ensuring product quality and reliability. However, PCB defects are often small, irregularly shaped, and embedded in complex textures, making them difficult to detect using traditional methods. In this paper, we propose CM-UNetv2, a semantic segmentation network designed to address these challenges through three architectural modules incorporating four key innovations. First, a Parallelized Patch-Aware Attention (PPA) module is incorporated into the encoder to enhance multi-scale feature representation through a multi-branch attention mechanism combining local, global, and serial convolutions. Second, we propose a Dual-Stream Skip Guidance (DSSG) module that decouples semantic refinement from spatial information preservation via two separate skip pathways, enabling finer detail retention. Third, we design a decoder module called Frequency-domain Guided Context Mamba (FGCMamba), which integrates two novel mechanisms: a Spatial Guidance Cross-Attention (SGCA) mechanism to enhance the alignment of spatial and semantic features, and a Frequency-domain Self-Attention Solver (FSAS) to compute global attention efficiently in the frequency domain, improving boundary restoration and reducing computational overhead. Experiments on the MeiweiPCB and KWSD2 datasets demonstrate that the CM-UNetv2 achieves state-of-the-art performance in small object detection, boundary accuracy, and overall segmentation robustness.

## 1. Introduction

With the increasing prevalence of electronic products, the manufacturing of printed circuit boards (PCBs) has become a fundamental aspect of modern industrial production [1]. As a core component of electronic devices, PCBs must exhibit excellent stability and anti-interference capabilities, while supporting high integration, compact design, and high-speed data transmission. However, even minor defects can lead to significant consequences, including reduced product quality, increased costs due to rework or scrap, delays in time-to-market, and potential safety risks such as short circuits, component failure, or overheating. As a result, extensive research has been conducted on PCB defect detection, and various algorithms have been proposed to improve both accuracy and efficiency.

Quality control is a critical aspect of the manufacturing process. To meet increasing market demands, it is essential to ensure stringent quality management and enhance production efficiency. Therefore, surface defect detection plays a critical role in enhancing quality in PCB manufacturing [2]. However, traditional defect detection methods [3,4], which rely primarily on manual visual inspection, suffer from low efficiency and high omission rates. As an alternative, computer vision-based surface defect detection methods [5,6] have gained significant attention due to their theoretical significance and practical value. In automatic detection methods for PCB surface defects, accurate segmentation of defect regions is especially crucial, as it produces binary maps that facilitate visual recognition of defects by computer systems.

The growing popularity of consumer electronics has significantly increased the demand for mass-produced, high-quality PCBs. However, maintaining such high quality during large-scale production poses major challenges. Current challenges in PCB image detection include inaccurate edge segmentation and missing regions in the segmentation of large-scale objects. Defects in PCBs can lead to circuit failure, making defect detections—specifically targeting the localization and classification of PCB defects—crucial. Developing efficient PCB defect detection methods not only improves manufacturing quality and efficiency but also plays an important role in advancing modern industrial production.

Therefore, we propose CM-UNetv2, a semantic segmentation network designed to enhance fine-grained structure extraction and robustness in complex scenarios. The architecture integrates a Parallelized Patch-Aware Attention (PPA) encoder for multi-scale feature extraction, a Dual-Stream Skip Guidance (DSSG) that processes two parallel feature streams—one refined through the MSAA module and the other directly passed to the decoder to retain original spatial structure details—and a Frequency-domain Guided Context Mamba (FGCMamba) decoder, which combines Frequency-domain Self-Attention (FSAS) with Spatial Guidance Cross-Attention (SGCA) to improve boundary and texture reconstruction. The main contributions of this work can be summarized as follows:

(1) To enhance feature representation in the encoding stage, we introduce the PPA module. It captures local and global context via multi-branch attention and produces three outputs for progressive encoding, semantic enhancement, and spatial skip connections.
(2) To address the balance of features in skip connections, we propose the DSSG mechanism. One path is processed through the MSAA module, while the other directly passes the original encoder features to the decoder.
(3) To integrate DSSG with the decoder, we introduce the FGCMamba Block. It processes both paths and combines refined semantic features with spatial details using SGCA, enabling precise fusion during decoding.
(4) To achieve a balance between modeling efficiency and accuracy, we incorporate the FSAS module into the FGCMamba decoder. This unified attention mechanism replaces traditional spatial and channel attention modules, thereby enhancing fine-grained feature representation while reducing computational complexity.

## 2. Relevant Works

### 2.1. PCB Defect Detection

Traditional computer vision techniques have achieved remarkable success in surface inspection applications [1,7,8]. For instance, ref. [9] introduced an automated defect cluster identification system for semiconductor wafers. In [10], researchers developed a hybrid two-stage one-versus-many support vector machine (SVM) approach for the automatic fault diagnosis of rolling bearings. The study in [2] utilized innovative multifractal features in conjunction with support vector data description to enable automated defect detection in fabrics.

With the widespread adoption of deep learning in vision tasks, these techniques have increasingly been applied to surface defect detection. For example, ref. [11] proposed an automatic defect detection strategy based on convolutional neural networks (CNNs) to improve the accuracy of identifying surface anomalies in workpieces. In [12], deep CNNs were used to recognize wood characteristics and automatically classify defects in images captured by laser scanners. A CNN integrated with attention mechanisms was introduced in [13] to accurately segment foreign objects in coal images with complex backgrounds. Study [6] combined Faster R-CNN [9] with dense scale-invariant features to detect defects in railway track fasteners. Ref. [14] further validated the effectiveness of deep learning in industrial visual inspection scenarios.

As deep learning continues to proliferate in computer vision, it has also been extensively applied to PCB (printed circuit board) surface defect detection. For example, Zhang et al. [15] proposed a deep feature learning-based approach for PCB bare board defect detection, leveraging a pretrained CNN to extract image features and using a sliding window for classification, significantly improving the accuracy of detecting scratches, disconnections, and other defects. Liu et al. [16] developed YOLO-pdd, a lightweight detection framework that combines the YOLOv5 backbone with multi-scale modules to achieve efficient detection in sequential images.

Additionally, Li et al. [17] presented an improved PCB defect detection model based on a feature pyramid network (FPN), which significantly enhanced multi-scale defect recognition. Another study [18] employed a denoising convolutional autoencoder for feature reconstruction and interpolation recovery of defect images, ultimately localizing defects through a difference map, achieving an overall accuracy of 97.5%. Meanwhile, Gustilo et al. [19] designed a lightweight CNN-based recognition network tailored for mobile devices. Using transfer learning to compensate for limited training data, their model achieved an 85% detection rate.

Despite their effectiveness, most existing PCB defect detection methods primarily focus on classification or coarse localization, lacking detailed information about the shape and boundaries of defects. This limits their usefulness in tasks requiring fine-grained analysis or accurate repair. In contrast, semantic segmentation provides pixel-level localization, enabling precise contour extraction and area measurement, making it more suitable for high-precision industrial applications.

### 2.2. Deep Learning-Based PCB Segmentation Methods

Surface defect segmentation plays a critical role in object detection and recognition tasks. Traditional approaches typically rely on handcrafted feature extraction combined with classifiers [20,21]. However, end-to-end deep learning approaches have recently achieved significant progress in semantic segmentation. Fully Convolutional Network (FCN) [22] is widely used; it replaces the fully connected layers in conventional CNNs with convolutional layers, allowing the model to accept input images of arbitrary size.

U-Net [23], a classic encoder–decoder architecture with skip connections, has demonstrated great success in medical image segmentation and has been adopted in various industrial applications. The DeeplabV3+ series [24] introduced atrous (dilated) convolutions to enlarge the receptive field while maintaining spatial resolution, effectively improving contextual information. PSANet [25] utilizes a pyramid pooling module to aggregate information across multiple scales. Moreover, attention mechanisms have been integrated to enhance feature representation. For instance, ref. [26] designed channel and spatial attention modules to enhance responses in important regions, while [27] applied contextual attention to model multi-scale context dependencies. Although these methods have shown strong performance on public benchmarks, their fixed receptive fields limit the capacity for global contextual modeling.

In recent years, semantic segmentation research focused on PCB surface defects has achieved notable progress. Unlike classification or object detection, semantic segmentation provides pixel-level localization, which is essential in industrial manufacturing scenarios where accurate shape, boundary, and location of defects must be determined.

Many researchers have proposed customized U-Net architectures for this purpose. For instance, Li and Liu proposed an improved U-Net with a VGG16 encoder, incorporating a dynamic hybrid attention module (DHAM) and a lightweight feature fusion module (RGSM). With only 22 million parameters, their model achieved 81.74% mIoU and 87.33% mPA while supporting 30 FPS inference, making it suitable for real-time applications [18]. Another study adopted denoising autoencoders to reconstruct input images and locate defects via difference maps, achieving effective unsupervised segmentation [28].

To further enhance global perception, researchers have incorporated Transformer architectures into segmentation tasks. Liu et al. combined a Swin Transformer backbone with a Cascade Mask R-CNN decoder and used Stable Diffusion to generate defect variants, significantly improving the detection and segmentation of small objects [29]. Similarly, PCB-DDTR (Detail and Dependency Transformer) proposed a dual-branch module combining local detail features and global context, effectively improving segmentation quality for complex defects [30]. The CA-TransSeg model introduced a change-aware Siamese Transformer network that fuses global contrast and change maps to detect subtle surface variations—ideal for pre/post-inspection tasks [31]. Lightweight design has also become a research focus. SEPDNet (Simple and Effective PCB Defect Network) achieves performance comparable to deeper models using fewer parameters, maintaining a good balance between speed and accuracy for high-resolution images [32]. However, scenarios with densely distributed micro-defects, blurred textures, and irregular fault patterns—especially under industrial noise like silk screen marks or oxidation—pose significant challenges for existing segmentation models. Such scenarios require improved preservation of spatial details and more adaptive semantic reasoning capabilities. Thus, reviewing recent advances in semantic segmentation is essential to identify more suitable approaches for such tasks.

### 2.3. Semantic Segmentation Methods

In the late 2010s, CNN-based methods became the dominant paradigm for semantic segmentation tasks [33]. To support medical image segmentation, Ronneberger et al. proposed the U-Net in 2015—an encoder–decoder structure where input images are downsampled via max pooling in the encoder and later combined with semantic features in the decoder for accurate boundary reconstruction [23]. In 2018, Zhou et al. introduced DLinkNet, which incorporated dilated convolutions to extract road features from remote sensing imagery and improve spatial connectivity [34]. That same year, Chen et al. proposed Deeplabv3+, which incorporated an Atrous Spatial Pyramid Pooling (ASPP) module using multiple dilation rates to capture features at various scales [24]. Overall, traditional CNN methods primarily depend on manually designed convolution kernels to integrate high-level and low-level features [35].

To reduce reliance on dense pixel-level annotations, several masked modeling strategies have been proposed for semantic segmentation. One representative method is Masked Supervised Learning [36], which randomly masks input patches during training and encourages the model to infer semantics from incomplete visual cues, thereby improving generalization. Masked Generative Distillation (MGD) [37] adopts a teacher-student framework in which the student network learns to reconstruct the teacher’s full feature representation from partially masked inputs, enhancing the feature quality for dense prediction tasks. Another approach, Masked Collaborative Contrast (MCC) [38], combines masked image modeling with contrastive learning by constructing positive pairs between masked local features and global semantic predictions, improving semantic consistency in weakly supervised settings. These methods represent valuable efforts to reduce annotation costs and improve label efficiency. However, most masked modeling strategies remain sensitive to the design of masking patterns and often assume strong spatial correlations within visible regions. This can lead to degraded performance when masked areas are semantically complex or large. 

Transformer-based models have subsequently gained popularity for their ability to capture global contextual relationships. For example, in 2021, Chen et al. proposed TransUNet, introducing the Vision Transformer module to learn self-correlations within feature maps using self-attention and assign adaptive weights to each pixel [39]. SegFormer [40] and SETR further simplified model architectures while maintaining hierarchical attention modeling capabilities. The Swin-Unet model extended this line of work by integrating Swin Transformer blocks within a U-Net architecture to preserve hierarchical spatial representations while benefiting from window-based self-attention [41]. However, Transformer-based models often incur high computational and memory costs, especially with high-resolution inputs. Their lack of locality bias can also limit the modeling of fine-grained structures, particularly for small or irregular objects.

The original Mamba model was developed for 1D sequence modeling using selective sequential scanning, particularly in natural language processing (NLP) tasks [42]. As interest in extending Mamba to vision tasks has grown, Xu et al. conducted a comprehensive survey highlighting both the promise and limitations of Mamba-based architectures in computer vision, particularly in modeling spatial and multi-scale structures [43]. To adapt Mamba to 2D image data, Liu et al. introduced the 2D Selective Scanning Module (2D-SSM) for image semantic segmentation in 2024 [44]. This module flattens image features and performs directional scanning to capture long-range dependencies and restore the 2D structure. Also in 2024, Zhao et al. proposed RS-Mamba, a neural network designed for remote sensing image segmentation and change detection. This model leveraged Mamba’s global modeling capabilities to enhance computational efficiency and segmentation quality [45]. In the same year, Liu et al. presented CM-UNet, which combines a ResNet encoder with a CSMamba module to enhance the fusion of global and local features [46]. Although these methods expand the receptive field beyond traditional CNNs, they often struggle to handle objects with irregular shapes and varying scales. Therefore, hybrid approaches combining CNN and Mamba modules should be considered.

## 3. Methodology

**Figure 1:**
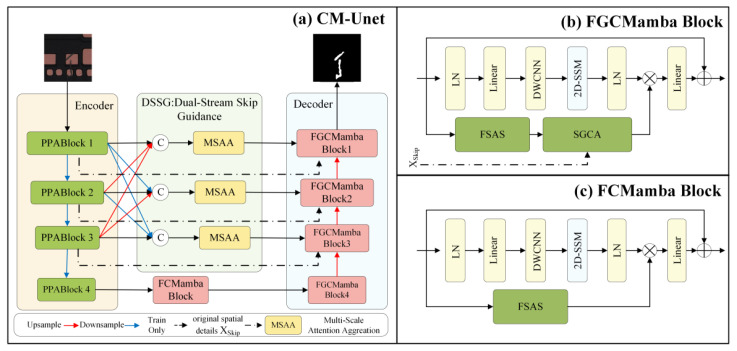
The structure of the proposed CM-UNetv2 network.

The CM-UNetv2 framework, as illustrated in Figure 1, consists of three main components: a PPA-based encoder, the DSSG module, and an FGCMamba decoder. The encoder incorporates the Parallelized Patch-Aware Attention (PPA) module to expand channels through parallel local and global branches, refining multi-scale features with attention mechanisms. At each resolution level, the PPA module generates two outputs: X and $X_{skip}$. In the DSSG module, $X_{skip}$, which retains the original spatial structure features, is directly passed to the decoder, while X is processed by the MSAA module, which enhances semantic features via multi-scale fusion and adaptive refinement. Both feature streams are subsequently input into the FGCMamba decoder, where the SGCA and FSAS modules effectively combine information from both paths, thereby improving segmentation accuracy and fine-detail reconstruction. Additionally, a lightweight FGCMamba module is placed between the encoder and decoder to simplify the attention mechanism by retaining only the frequency-domain self-attention component, thus improving computational efficiency.

### 3.1. Parallelized Patch-Aware Attention Module

Traditional CNNs such as ResNet increase the receptive field via repeated downsampling, but this often causes loss of fine-grained structural details. To address this issue, we introduce the PPA module into the encoder. This module enhances multi-scale representation through a parallel multi-branch architecture and integrated attention mechanisms.

**Figure 2:**
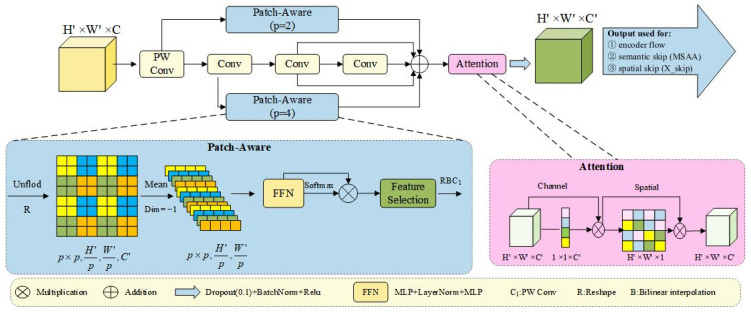
The module consists of two main components: a multi-branch feature fusion mechanism and a sequential attention refinement. The fusion stage combines patch-aware branches (with patch sizes p=2 and p=4 for local and global context) with a serial convolutional branch to enhance multi-scale representation. The aggregated features are further refined using channel and spatial attention modules. The output is split into three pathways for progressive encoding, semantic refinement, and spatial skip connection, as illustrated.

The core of PPA lies in its multi-branch feature extraction strategy. As shown in Figure 2, given the input feature $F\in R^{H^{\prime }\times W^{\prime }\times C^{\prime }}$, a point-wise convolution produces the adjusted feature $F^{\prime }\in R^{H^{\prime }\times W^{\prime }\times C^{\prime }}$. Then, three parallel branches-local, global, and serial convolutions-extract feature $F_{\mathrm{local}}$, $F_{\mathrm{global}}$, $F_{\mathrm{conv}}\in R^{H^{\prime }\times W^{\prime }\times C^{\prime }}$, which are summed element-wise to form the fused representation $\tilde{F}\in R^{H^{\prime }\times W^{\prime }\times C^{\prime }}$.

In the local and global branches, we enhance receptive field diversity by adjusting the patch size p and performing patch-wise processing on the input feature $F^{\prime }$. Specifically, $F^{\prime }$ is divided into non-overlapping patches of size p×p patches, forming a tensor of shape (p×p, $H^{\prime }/p$, $W^{\prime }/p$, C). Channel-wise average pooling reduces this to (p×p, $H^{\prime }/p$, $W^{\prime }/p$), followed by a feed-forward network FFN [47] and activation function to compute spatial attention weights.

To enhance feature selection, we introduce a cosine similarity-based token reweighting mechanism. Let $d=H^{\prime }\times W^{\prime }/(p\times p)$, and denote the tokens as ${\left(t_{i}\right)}_{i=1}^{C^{\prime }}$, where $t_{i}\in R^{d}$. Each token is reweighted by its similarity to a task embedding $\xi \in R^{C^{\prime }}$, as follows:

(1) $${\overset{\wedge }{t}}_{i}=P\cdot sim(t_{i},\xi )\cdot t_{i}$$

where $P\in R^{C^{\prime }\times C^{\prime }}$ is a learnable projection matrix and sim(⋅) denotes normalized cosine similarity.

After reweighting, channel selection is performed on each token, followed by reshape and interpolation to restore the original spatial dimensions, yielding local and global features $F_{\mathrm{local}}$ and $F_{\mathrm{global}}$, both of shape $R^{H^{\prime }\times W^{\prime }\times C^{\prime }}$. The convolutional branch applies three serial 3 × 3 convolutions, and their outputs ($F_{\mathrm{conv}1}$, $F_{\mathrm{conv}2}$, $F_{\mathrm{conv}3}$) are summed to obtain $F_{\mathrm{conv}}\in R^{H^{\prime }\times W^{\prime }\times C^{\prime }}$.

The fused feature $\tilde{F}\;$is then upsampled to $R^{H\times W\times C^{\prime }}$ and refined using a sequential attention mechanism. A channel attention map $M_{c}\in R^{1\times 1\times C^{\prime }}$ and a spatial attention map $M_{s}\in R^{H^{\prime }\times W^{\prime }\times 1}$ are applied as follows:

(2) $$F_{c}=M_{c}(\overset{\sim }{F})⊗\overset{\sim }{F}$$

(3) $$F_{S}=M_{s}(F_{c})⊗F_{c}$$

(4) $$F\prime \prime =\delta (\beta (dropout(F_{s})))$$

where ⊗ denotes element-wise multiplication, δ is ReLU, $\beta \left(\cdot \right)$ represents batch normalization, and $F^{\prime \prime }\in R^{H\times W\times C^{\prime }}$ is the final output of PPA.

In our implementation, the output of the PPA module is configured to support three distinct pathways: one branch is passed to the next PPA layer to maintain progressive encoding; the second serves as a semantic refinement stream into the MSAA module; and the third acts as a spatial-detail-preserving skip connection $X_{skip}$ for decoder integration. This output strategy enables effective dual-stream guidance in the proposed DSSG module while ensuring continuity across encoder layers.

### 3.2. Dual-Stream Skip Guidance

CM-UNet adopts a Multi-Scale Attention Aggregation (MSAA) module that dynamically fuses encoder features at each decoding stage, injecting rich global semantic information into skip connections and enhancing the model’s ability to interpret complex scenes. The MSAA module refines spatial and semantic representations by aggregating multi-scale features, allowing the model to better capture both fine-grained details and broader contextual information, which is essential for accurate segmentation. However, fusing features before transmission tends to weaken spatial details. While this fusion enhances semantic representation, it compromises spatial precision, particularly in tasks where fine details are critical, such as small object detection.

To address this issue, we propose a novel dual-path skip connection architecture, named the Dual-Stream Skip Guidance (DSSG). This module explicitly decouples semantic enhancement from spatial preservation by leveraging the dual-output capability of the PPA module. Specifically, each PPA block produces two separate streams: a semantically enriched representation **X**, and a high-resolution spatial stream $X_{skip}$ In the DSSG pathway, **X** is passed through the MSAA module for multi-scale refinement, while $X_{skip}$ bypasses semantic transformation and is directly forwarded to the decoder, preserving fine-grained spatial structure.

From a modeling perspective, DSSG establishes an explicit bifurcation of the representational space, encouraging the encoder to separate semantic abstraction and spatial preservation into orthogonal pathways. Formally, the outputs of a PPA block can be denoted as follows:

(5) $$X\in MSAA\left(F\right),\;\;X_{skip}=F$$

where F is the feature tensor produced by PPA. This formulation enforces a task-specific inductive bias that aligns with the principles of representation disentanglement, allowing the model to assign complementary roles to different branches. As a result, semantic refinement does not overwrite location-sensitive details, which is crucial for accurate segmentation in texture-rich or cluttered environments.

These two streams are subsequently processed within the FGCMamba decoder. They are integrated via the Spatial Guidance Cross-Attention (SGCA) mechanism, where **X** serves as the query and $X_{skip}$ provides the key and value. This guided fusion allows the decoder to selectively emphasize salient semantic cues while anchoring predictions to spatial structure, enhancing segmentation accuracy and boundary localization.

### 3.3. Frequency-Domain Guided Context Mamba Block

Leveraging the DSSG proposed in Section 3.2, we enhance the decoder by introducing the Frequency-domain Guided Context Mamba Block (FGCMamba Block), which integrates two mechanisms: Spatial Guidance Cross-Attention (SGCA) and a Frequency-domain Self-Attention Solver (FSAS). These components aim to enhance multi-dimensional feature modeling.

The first enhancement of the FGCMamba block is the Spatial Guidance Cross-Attention (SGCA) mechanism, which explicitly models the interaction between semantic and spatial representations. In this setup, the semantic feature stream **X**, refined through the MSAA module, is used as the query, while the spatial stream $X_{skip}$, which retains raw encoder details, serves as both key and value.

This configuration allows SGCA to compute attention weights that reflect the alignment between high-level semantic patterns and low-level spatial structures. By structuring the interaction in a query–key–value framework, the model performs a projection of semantic content onto spatial geometry, enabling feature correlation across representational domains. Unlike standard self-attention, this design enforces a directional flow from semantic abstraction toward spatial anchoring, which aligns with the principle of cross-modal attention and supports disentangled representation learning.

**Figure 3:**
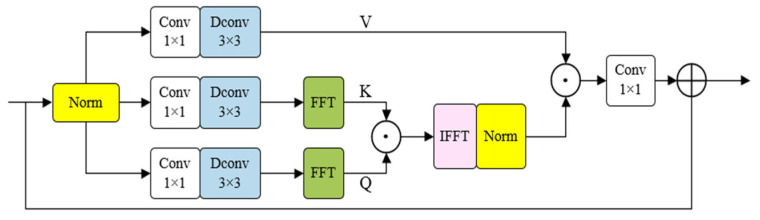
The proposed FSAS module. Q (query), K (key), and V (value) are derived from the input feature via 1 × 1 and 3 × 3 convolutions. Q and K are processed in the frequency domain to compute attention, and V is used to aggregate contextual information.

The second improvement replaces the separate spatial and channel attention with the FSAS, which replaces conventional spatial-domain attention with a frequency-aware formulation. Instead of computing attention maps directly in the spatial domain, FSAS leverages the Fourier transform to capture long-range dependencies more efficiently. As shown in Figure 3, Given input features ${F\in R}^{H\times W\times C}$, FSAS first generates three projections via 1×1 convolutions:

(6) $$F_{q}=C\mathrm{on}v_{1\times 1}\left(F\right),F_{k}=C\mathrm{on}v_{1\times 1}\left(F\right),F_{v}=C\mathrm{on}v_{1\times 1}\left(F\right)$$

Then, attention is computed in the frequency domain. Fast Fourier Transforms (FFT) are applied to $F_{q}$ and $F_{k}$, and their spectral correlation is obtained via element-wise complex multiplication:

(7) $$A=F^{-1}(F(F_{q})\cdot \bar{F(F_{k})})$$

where $F\left(\cdot \right)$ and $F^{-1}\left(\cdot \right)$ denote Fourier and inverse Fourier transforms, · indicates complex multiplication and $\bar{\left(\cdot \right)}$ represents conjugation. The attention map A is normalized and multiplied by $F_{v}$. Finally, a 1×1 convolution and residual connection form the output:

(8) $$Y=C\mathrm{on}v_{1\times 1}(F_{v}\cdot LayerNorm(A))+X$$

The frequency-domain formulation allows FSAS to efficiently capture global contextual relationships while reducing computational costs. Compared to traditional spatial attention, it achieves a better trade-off between expressiveness and efficiency, improving fine-grained perception. Finally, the outputs of the main processing path and the frequency-aware gating path are combined through an element-wise modulation mechanism. By adaptively weighting decoded feature maps with gated signals derived from SGCA and FSAS, FGCMamba enables selective emphasis of informative regions, achieving a balanced integration of semantic context and spatial detail, and substantially enhancing segmentation accuracy.

### 3.4. Loss Function

We mainly use the basic cross-entropy and Dice methods as loss functions because all the masks in our datasets consist of two classes, namely a single target and the background.

(9) $$L_{BCE+Dice}=\lambda_{1}L_{BCE}+\lambda_{2}L_{Dice}$$

(10) $$L_{BCE}=-\frac{1}{N}\sum_{N}^{1}\left[y_{i}log({\hat{y}}_{i})+(1-y_{i})\log(1-{\hat{y}}_{i})\right]$$

(11) $$L_{Dice}=1-\frac{2\left|X\cap \left.Y\right|\right.}{\left|X\right|+\left|Y\right|}$$

Among them, $\lambda_{1}$ and $\lambda_{2}$ are constants, and (1, 1) is usually selected as the default parameter.

## 4. Experiments

### 4.1. Datasets

To comprehensively evaluate the generalizability and cross-task robustness of the proposed method in different application fields, we selected two publicly available datasets that differ significantly in characteristics, representing two typical scenarios: industrial surface defect detection and remote sensing semantic segmentation. These two datasets are highly complementary in terms of image resolution, target morphological complexity, texture features, and background interference, and can effectively evaluate the model’s structural representation capabilities and generalization performance.

#### 4.1.1. MeiweiPCB Dataset

**Figure 4:**
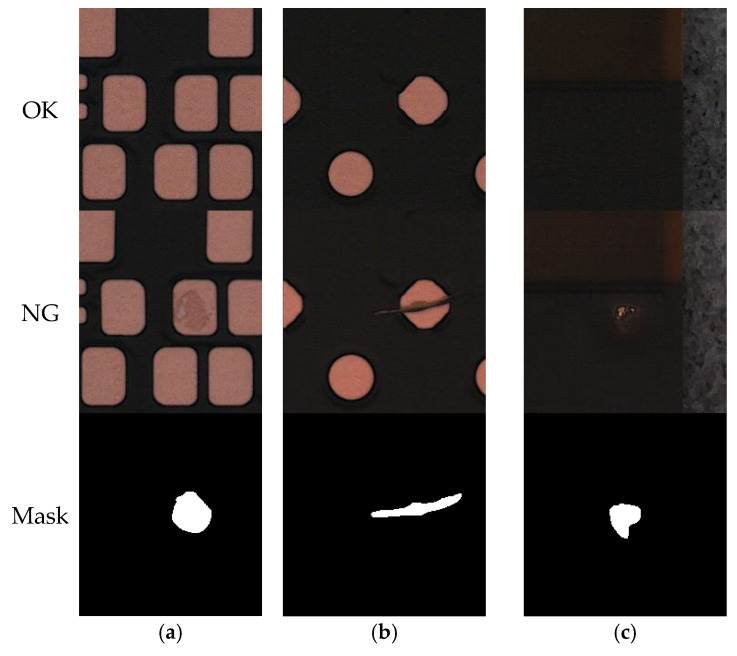
Visualization examples of the MeiweiPCB dataset: (**a**) surface contamination: normal image, defect image, and mask; (**b**) scratch defect: normal image, defect image, and mask; and (**c**) foreign object intrusion: normal image, defect image, and mask.

This study uses the MeiweiPCB segmentation dataset, whose annotation information has been publicly released on the GitHub platform (https://github.com/youtang1993/MeiweiPCB, accessed and used based on the version released on 6 May 2025). Developed by Meiwei Electronics, MeiweiPCB is a high-quality printed circuit board (PCB) defect detection dataset acquired from real industrial production lines. It is specifically designed for surface defect segmentation tasks. As illustrated in Figure 4, the dataset contains 926 images captured by industrial line-scan cameras, all cropped into uniform patches of 220 × 220 pixels. It encompasses typical industrial defect types, including Open Circuit, Burr, Missing Hole, and Excess Copper, with each defective image accompanied by high-quality pixel-level semantic segmentation labels. Due to the complex background texture, significant grayscale variations, and interference from imaging noise, illumination variation, and occlusion, this dataset presents substantial challenges for boundary modeling and detail preservation. For experimental consistency, all images were zero-padded to 256 × 256 pixels and split into a training set (80%) and a test set (20%) for model training.

#### 4.1.2. KWSD2 Water Body Segmentation Dataset

**Figure 5:**
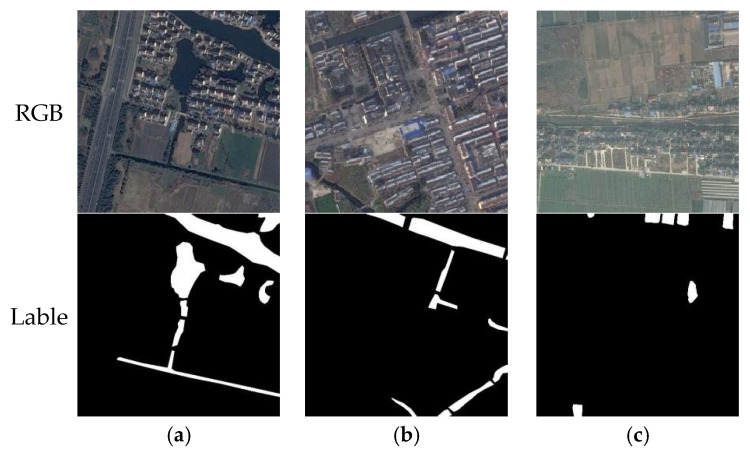
Visualization examples of the KWSD2 dataset: (**a**) water bodies near roads; (**b**) water bodies near residential areas; and (**c**) small ponds in urban areas.

This study uses the Kaggle Water Segmentation Dataset 2 (KWSD2), with annotation information that has been publicly released on the Kaggle platform (https://www.kaggle.com/datasets/kaoyuu/kaoyuuu/data, accessed on 3 April 2025). KWSD2 (KaoYuuu Water Segmentation Dataset) is a benchmark dataset in the field of remote sensing image analysis, specifically designed for high-precision extraction of surface water body areas. As illustrated in Figure 5, the dataset contains high-resolution remote sensing images from different geographical locations and time phases, with rich image contents and large geomorphic changes, covering a wide range of water body forms such as lakes, rivers, reservoirs, and wetlands.

The images involve complex background factors such as vegetation interference, water surface reflection, and cloud occlusion, along with drastic changes in target structure morphology and blurred boundaries, posing great challenges to the model’s context awareness and edge restoration. This dataset serves as an important benchmark for evaluating the performance of remote sensing semantic segmentation models, particularly in terms of long-range dependency modeling, spatial structure preservation, and cross-scale feature integration.

The original resolution of the images is 492 × 492. To adapt to the input of neural networks, we padded 10 pixels around each image, resulting in standardized input samples of 512 × 512 pixels. In the preprocessing stage, we binarized the label maps: pixels with gray values greater than 127 were regarded as the “water body” category, and the rest were regarded as the “non-water body” category. Eventually, the entire dataset was divided into 800 training images and 200 test images for supervised learning and performance evaluation.

### 4.2. Experimental Details and Evaluation Metrics

The experiments were conducted using the PyTorch 2.0.1 framework on an Ubuntu 20.04 operating system with CUDA 11.8 support. We employed the AdamW optimizer with an initial learning rate of 5 × 10^−5^, which was decayed exponentially after each step. Data augmentation techniques such as random flipping and rotation were applied during training. The batch size was set to 8, and the maximum number of training epochs was 100. To comprehensively evaluate model performance, we adopted Precision, Recall, F1-score, and mIoU as evaluation metrics.

### 4.3. Experimental Results

The proposed CM-UNetv2 network demonstrates excellent performance on both MeiweiPCB and KWSD2 datasets. As summarized in Table 1, the model achieves an mIoU of 0.8224 on the MeiweiPCB dataset and 0.9516 on the KWSD2 dataset, outperforming existing mainstream methods. Additionally, the model demonstrates high consistency between Precision and Recall values, with minimal difference between the two, verifying the stability and robustness of the model in different types of target detection.

**Figure 6:**
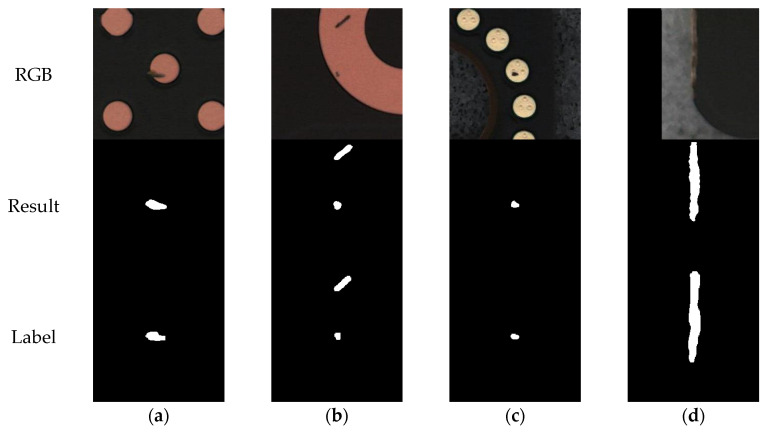
Examples of defect segmentation results of CM-UNetv2 on the MeiweiPCB dataset: (**a**) small defect at the center of a via; (**b**) two independent defects distributed separately; (**c**) small defect with blurred edges and interference from approximate circles; and (**d**) longitudinal strip-shaped defect in the edge region of the image.

Figure 6 illustrates representative segmentation results from the MeiweiPCB dataset. In Figure 6a, a small defect at the center of a via is correctly segmented, with contours closely matching the ground truth. Figure 6b illustrates accurate identification and separation of two distinct defects. Figure 6c presents a case involving a defect with blurred edges inside a circular pad, surrounded by similar circular structures; the model precisely localizes the defect region. Figure 6d demonstrates the model’s ability to identify a thin strip-shaped defect near the image edge, even under strong background texture interference.

**Figure 7:**
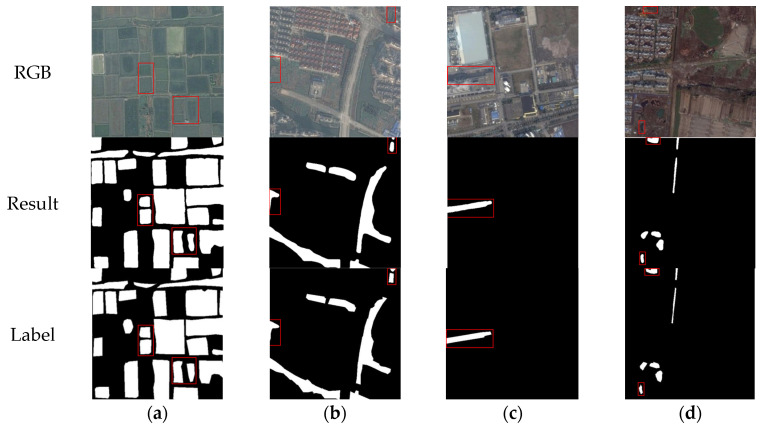
Examples of water body segmentation results of CM-UNetv2 on the KWSD2 dataset: (**a**) agricultural area. The red boxes clearly demonstrate the model’s ability to distinctly segment adjacent water bodies; (**b**) urban region. Despite complex surroundings and blurred boundaries, the red boxes show that the model accurately delineates water edges and preserves continuity; (**c**) industrial zone. The long and narrow water structures are successfully segmented with smooth contours, demonstrating strong shape preservation ability; and (**d**) suburban environment. The model accurately detects scattered water patches and narrow streams under cluttered backgrounds, as shown in the highlighted areas.

Figure 7 demonstrates the water body segmentation effect of our method on the KWSD2 dataset. As shown in Figure 7a, farmland water bodies with regular distribution and small ponds are successfully extracted. Figure 7b shows successful segmentation in low-contrast conditions between water and surrounding vegetation. Figure 7c shows the model’s effect of successfully extracting water bodies in urban scenes. Figure 7d presents the segmentation performance of the model in urban–rural junction areas, where the model can accurately identify the main water body structures, especially successfully extracting the slender irrigation canals.

### 4.4. Ablation Study

To comprehensively evaluate the effectiveness of each component in the proposed model, we designed two groups of ablation experiments to systematically verify the contribution of key modules to the overall performance from different perspectives.

The first group of experiments evaluated three core modules: the improved PPA module, the DSSG, and the FSAS-based FGCMamba module in the decoder. We sequentially removed or replaced each module and compared their performance through metrics such as mIoU, F1 score, and Precision, aiming to analyze the roles of each module in contextual feature modeling, spatial detail preservation, and frequency-domain feature enhancement.

The second group of experiments focuses on evaluating performance and efficiency under different configurations of embedding dimensions and network depth. Multiple architectures were constructed with varying embedding sizes and depths, compared under consistent training settings, and the impact of these factors was analyzed on overall performance.

The results from both experimental groups validate the independent contributions and synergistic effects of each module, further demonstrating the effectiveness and versatility of the proposed CM-UNetv2 architecture in various segmentation tasks.

#### 4.4.1. Network Architecture

As shown in Table 2 and Table 3, Modules A, B, and C all bring significant improvements in key metrics such as mIoU and Precision, demonstrating their effectiveness in feature extraction, boundary modeling, and detail restoration.

**Figure 8:**
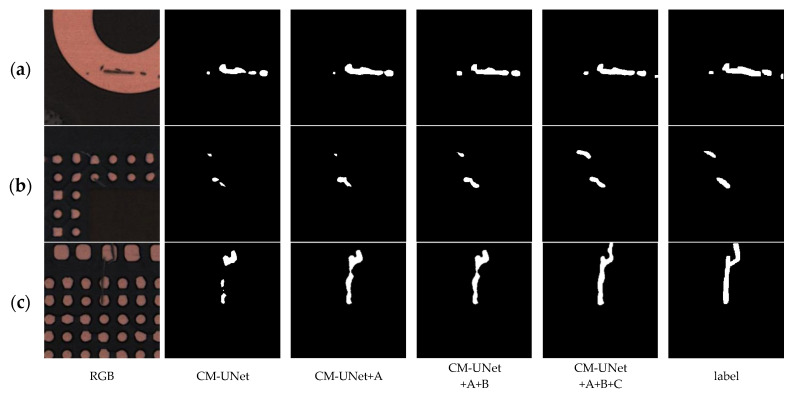
Examples of results from the ablation study on network structure on the MeiweiPCB dataset: (**a**) the PPA module enhances the perception of elongated scratches, reconnecting fragmented regions and improving continuity; (**b**) DSSG further improves the detection of small-scale defects in cluttered areas by preserving spatial detail and suppressing noise; and (**c**) with all three modules integrated, the model achieves the most accurate segmentation, successfully recovering long and narrow regions with high boundary fidelity.

**Figure 9:**
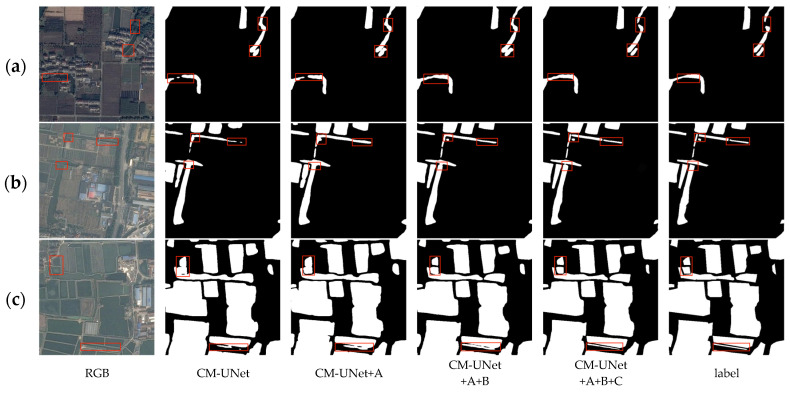
Examples of ablation study results on network structure using the KWSD2 dataset. (**a**) The red boxes in the first example highlight adjacent narrow rivers; only with the complete model does the segmentation maintain clear separation, indicating that the PPA module is essential for distinguishing fine-grained structures. (**b**) In the second example, the red boxes show regions with boundary ambiguity and interference, where the inclusion of the DSSG module helps refine object contours and reduce over-segmentation. (**c**) In the third case, the addition of the FSAS module enables better recovery of long and fragmented water bodies, enhancing global consistency and restoring overall structural completeness.

Module A introduces the Patch-wise Patch Attention (PPA) mechanism, which enhances the model’s ability to capture multi-scale contextual information and represent fine-grained structures. On the KWSD2 dataset, mIoU increases to 0.9298 and Precision to 0.9540; on the MeiweiPCB dataset, mIoU increases to 0.8061 and Precision to 0.8680. As illustrated in Figure 8a and Figure 9b, PPA significantly improves the model’s perception of elongated river-like structures and fine details, successfully reconnecting some broken regions, though it also introduces slight over-expansion in certain areas.

Module B (DSSG) adopts a dual-branch architecture: one branch passes through the MSAA module for semantic enhancement, while the other directly transmits spatial features to the decoder FGCMamba to retain detailed information. At this stage, FGCMamba does not incorporate the FSAS module, but utilizes SGCA to guide the fusion between encoder and decoder features for more accurate boundary modeling. On the KWSD2 and MeiweiPCB datasets, mIoU reaches 0.9336 and 0.8099, respectively. As shown in Figure 8b and Figure 9c, the addition of DSSG on top of PPA further suppresses redundant predictions, sharpens boundary contours, and aligns predicted structures more closely with actual targets.

Module C introduces the FSAS module, which leverages frequency-domain attention to enhance the model’s responsiveness to critical regions while effectively suppressing background noise, thereby improving fine-detail representation. On the KWSD2 and MeiweiPCB datasets, mIoU improves to 0.9250 and 0.7978, respectively. As seen in Figure 8c and Figure 9a, FSAS strengthens long-range dependency modeling, leading to more accurate boundary recovery and smoother, more precise contours.

Finally, when all three modules (A+B+C) are used together, the model achieves the best performance, with mIoU reaching 0.9516 on KWSD2 and 0.8224 on MeiweiPCB. As shown in Figure 8c and Figure 9a, the model demonstrates superior capability in boundary structure preservation, detail restoration, and noise suppression. PPA enhances sensitivity to defect regions, DSSG improves semantic–spatial decoupling and reduces over-segmentation, while FSAS further optimizes global consistency and boundary accuracy.

#### 4.4.2. Embedding–Depth Architecture

To explore the impact of embedding dimensions (Embed Dim) and network depths (Depths) on segmentation performance, we conducted ablation experiments using three configurations of CM-UNetv2 on the KWSD2 and MeiweiPCB datasets. The input resolutions were set to 512 × 512 for KWSD2 and 256 × 256 for MeiweiPCB.

As presented in Table 4, on the MeiweiPCB dataset, which presents finer textures and diverse defect types, overall performance was slightly lower due to the increased complexity of industrial defect segmentation. All configurations achieved mIoU values above 0.81, but again, the 96/2 configuration stood out. It attained the highest precision (0.8970), recall (0.8882), F1-score (0.8925), and mIoU (0.8224). These results highlight its robustness in preserving fine-grained features under noisy and occluded conditions. The 128/3 configuration delivered consistent results (Precision: 0.8944, Recall: 0.8850, F1-score: 0.8897, mIoU: 0.8199), while the 64/4 configuration lagged slightly with a lower mIoU of 0.8178, despite similar precision and recall levels (0.8886 and 0.8840, respectively). This indicates that increased depth alone may not compensate for reduced embedding richness when fine detail representation is critical.

As shown in Table 5, on the KWSD2 dataset, all three configurations achieved strong results, but subtle differences were observed. This dataset includes high-resolution remote sensing images characterized by complex structures, blurred boundaries, and background interference, which require strong global context modeling and fine boundary delineation. Among the three configurations, the model with an embedding dimension of 96 and depth of 2 achieved the highest overall performance, with a precision of 0.9632, recall of 0.9720, F1-score of 0.9676, and mIoU of 0.9516. This reflects a balanced ability to capture semantic context and spatial detail. The configuration with an embedding dimension of 128 and depth of 3 also performed competitively (Precision: 0.9618, Recall: 0.9705, F1-score: 0.9661, and mIoU: 0.9505), showing only slight reductions in F1 and mIoU. Meanwhile, the 64/4 configuration yielded the lowest mIoU (0.9495) despite strong precision and recall (0.9602 and 0.9690, respectively), suggesting that deeper architectures with shallow embeddings may be suboptimal for capturing global and fine-grained features simultaneously.

In summary, the configuration using an embedding dimension of 96 and a network depth of 2 demonstrated the best generalization across both datasets. It offered an optimal trade-off between structural simplicity and representational power, making it suitable for both large-scale scene segmentation and fine-grained industrial defect detection. These results highlight the importance of carefully balancing embedding richness and network depth to enhance the segmentation model’s accuracy, generalization, and robustness.

### 4.5. Comparative Study with Other Methods

In this section, we compare the proposed method with other semantic segmentation neural networks on the MeiweiPCB and KWSD2 datasets to validate its overall effectiveness and robustness.

#### 4.5.1. Comparative Study with Other Methods on MeiweiPCB

**Figure 10:**
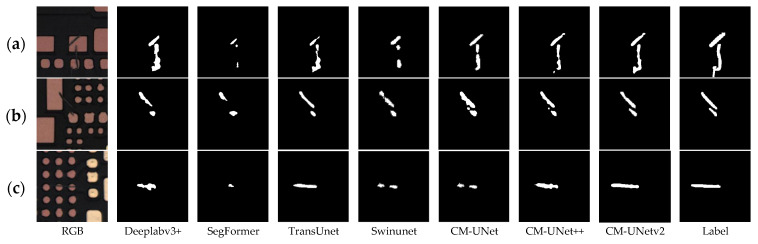
Examples of comparative results with other semantic segmentation methods on the MeiweiPCB dataset: (**a**) in low-contrast scenarios where defects are close in color to the background, have blurred edges, and obvious texture interference, CM-UNetv2 demonstrates stronger detail recognition capabilities; (**b**) in scenarios with clear defect boundaries and high contrast, CM-UNetv2 still maintains the optimal overall restoration effect, with scratch shapes highly consistent with ground-truth labels; and (**c**) under complex background interference where subtle scratches are obscured by dense structures, CM-UNetv2 successfully extracts complete defect regions by virtue of its good anti-interference ability.

In this subsection, we compare the proposed method with other semantic segmentation neural networks on the MeiweiPCB and KWSD2 datasets to validate its effectiveness. To verify the effectiveness of the proposed method for this task, we compared it with other state-of-the-art methods in semantic segmentation, including DeeplabV3+ [24], SegFormer [40], TransUNet [39], SwinUNet [41], CM-UNet [46], and CM-UNet++ [48]. As shown in Table 6, CM-UNetv2 performs optimally in Recall, F1-score, and mIoU metrics, with TransUNet and CM-UNet++ ranking second and third, respectively—both outperforming the other baseline models. As shown in Figure 10, CM-UNetv2 outperforms other methods in multiple defect detection scenarios on the MeiweiPCB dataset. Both CM-UNetv2 and TransUNet demonstrate good overall structural restoration, with CM-UNetv2 having more advantages in boundary accuracy and detail preservation. In Figure 8a, for subtle scratches with extremely low contrast, blurred edges, and texture similarity to the background, only CM-UNetv2 successfully segments the defect region completely and continuously. Although TransUNet and CM-UNet++ also detect the main areas, there are missing and incoherent issues, while SegFormer and SwinUNet fail to detect low-contrast regions effectively, only identifying limited high-contrast segments. Figure 8b shows a scratch scenario with high contrast, where the overall detection difficulty is relatively low and most methods can identify the main defect structure. However, in terms of boundary restoration, CM-UNetv2 performs the best, and the segmentation results are highly consistent with the ground truth labels. TransUNet and CM-UNet++ capture the complete main body, but there are still missing details at the edges. CM-UNet performs slightly worse with slight adhesion, while DeepLabv3+, SwinUNet, and SegFormer have missed detections and failed to cover the complete defect. The scratches in Figure 8c are located in the dense via array with a complex background, which puts forward higher requirements for the model’s anti-interference ability. Only CM-UNetv2 accurately detects the entire scratch with well-preserved boundary clarity, even in the presence of strong texture interference. TransUNet and CM-UNet++ can recognize the main structure, but the edges are broken or slightly offset; CM-UNet performs moderately, and some regions fail to separate from the background. In various challenging scenarios, CM-UNetv2 demonstrates the most stable and comprehensive performance, especially showing stronger robustness and detail preservation under complex backgrounds and low-contrast defects.

#### 4.5.2. Comparative Study with Other Methods on the KWSD2 Dataset

**Figure 11:**
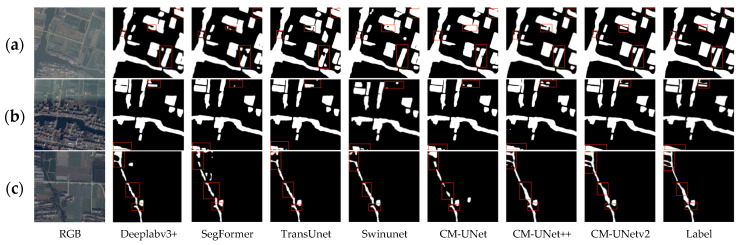
Examples of comparative results with other semantic segmentation methods on the KWSD2 dataset: (**a**) in scenarios where the texture difference between water bodies and the background is small and multiple adjacent water areas exist, CM-UNet++ can extract water structures more accurately; and (**b**) when there are multiple narrow water bodies affected by occlusion in urban areas, CM-UNetv2 outperforms other methods in segmentation, enabling more complete extraction of water structures; (**c**) in scenes where the contrast between irrigation canals and background fields is indistinct and water bodies are narrow, CM-UNetv2 better maintains the integrity of water structures compared to other methods, with segmentation results closer to ground truth labels.

To verify the generalization ability of our method across different task scenarios, we conducted experiments on the KWSD2 dataset using the same approach as in Section 4.5.1. Except for the input features, all hyperparameters were consistent with the MeiweiPCB dataset. For all methods mentioned in Section 4.5.1, the results in Table 7 show similar trends to those in Table 6. The proposed CM-UNetv2 achieves the best performance among these methods, ranking first in Recall, F1-score, and IoU metrics, with its mIoU exceeding that of other methods by over 1%, demonstrating strong segmentation accuracy in complex remote sensing scenarios. In the Precision metric, CM-UNetv2 ranks second with a score of 0.9486—0.84% higher than DeeplabV3+’s 0.9402 and superior to all other methods—further confirming its comprehensive effectiveness. As illustrated in Figure 11a, in scenarios with complex water body shapes, irregular boundaries, and multiple adjacent water areas, only CM-UNetv2 successfully extracts all water regions with complete and unbroken structures. CM-UNet++ follows closely, accurately segmenting most water areas with good boundary processing, but fails to detect some small isolated areas. Figure 11b depicts a mixed urban building and water network area containing multiple narrow, irregularly distributed water bodies, with some regions interfered with building shadows, posing high segmentation difficulty. In this scenario, CM-UNetv2 performs optimally, capable of relatively complete extraction of main water structures with better contour consistency. Figure 11c shows a typical rural scene with interlaced fields and irrigation canals, where water bodies are narrow with large width variations and low contrast with surrounding backgrounds (e.g., roads, fields, vegetation). In this image, CM-UNetv2 delivers the most accurate and complete segmentation results, extracting the majority of water structures with minimal discontinuities and boundaries closely matching the ground truth. CM-UNet++ also performs well with clear main water structures, but has interruptions in some branches. DeeplabV3+ has certain structural restoration ability but lacks boundary precision. TransUNet and CM-UNet show missed segmentation and fractures, while SwinUNet and SegFormer exhibit obvious incoherent segmentation in narrow canals with poor water structure integrity.

#### 4.5.3. Computational Complexity Comparison

Table 8 summarizes the performance of segmentation models across four key metrics: FLOPs, parameter count, inference speed (FPS), and mIoU. CM-UNetv2 achieves the highest mIoU (0.8224) while maintaining relatively low computational cost (21.63 GFLOPs) and a moderate parameter count (22.02 M), demonstrating strong capability in semantic representation and boundary localization. In contrast, TransUNet incurs a heavy parameter load (93.23 M), limiting its suitability for deployment in resource-constrained industrial settings. CM-UNet++, though extremely lightweight in parameters (8.71 M), adopts densely nested skip connections that lead to significant computational overhead (71.65 GFLOPs) and reduced inference speed (36.43 FPS). To address these issues, CM-UNetv2 incorporates a more efficient Dual-Stream Skip Guidance (DSSG) mechanism, which decouples semantic refinement and spatial preservation, allowing spatial features to bypass unnecessary transformations. This design minimizes redundant operations and effectively reduces FLOPs while retaining fine-grained detail. In addition, the FSAS module enhances global perception and boundary sensitivity via lightweight frequency-domain attention, further improving segmentation quality with minimal computational cost. Combined, these components enable CM-UNetv2 to achieve a superior balance between accuracy and efficiency, making it well suited for industrial segmentation tasks with tight performance and deployment constraints.

To further analyze the contributions of each component, we conducted internal ablation studies at both 256 × 256 and 512 × 512 input resolutions. As shown in Table 9, the results clearly identify the PPA module as the computational core of the model. At the 256 × 256 resolution, removing PPA leads to the most significant reduction in complexity—FLOPs decrease by 6.44 G, parameters are reduced by 7.71 M, and the inference speed increases markedly to 56.12 FPS. However, as demonstrated in the structural ablation experiments in Section 4.4, the PPA module is indispensable for achieving high segmentation accuracy and reconstructing fragmented targets. Its Patch-wise Patch Attention mechanism equips the model with strong multi-scale feature modeling and spatial structural awareness, making its performance contribution irreplaceable despite its higher computational cost.

In contrast, the DSSG and FSAS modules serve as lightweight yet effective enhancement components. The DSSG module introduces a dual-stream pathway that facilitates both semantic enrichment and spatial detail preservation, thereby improving boundary modeling accuracy with minimal computational overhead. Meanwhile, the FSAS module leverages a frequency-domain attention mechanism to enhance the model’s responsiveness to critical regions, effectively suppressing background noise and improving detail recovery. Table 9 and Table 10 demonstrate that ablating either DSSG or FSAS results in only minor changes in FLOPs, further confirming their lightweight design. In conjunction with PPA, these modules collaboratively refine boundary delineation and detail representation on top of the robust semantic foundation established by PPA.

Finally, we assess the model’s scalability under high-resolution input. The relative computational cost of each module remains consistent across different input sizes, validating the stability and robustness of our architectural design. In summary, PPA forms the core of CM-UNetv2’s representational capacity, while DSSG and FSAS provide crucial and efficient fine-grained enhancements. This collaborative design enables our model to achieve an optimal balance among accuracy, inference speed, and computational cost, demonstrating its strong potential for wide deployment in industrial applications.

## 5. Discussion

As demonstrated in Section 4.5.1 and Section 4.5.2, the proposed CM-UNetv2 network exhibits leading comprehensive performance in two representative task scenarios: industrial defect detection (MeiweiPCB dataset) and remote sensing water body segmentation (KWSD2 dataset). This superior performance primarily benefits from the model’s systematic architectural enhancements and introduction of key modules, including the MSAA dual-path skip connection mechanism (DSSG), PPA encoder, FGCMamba decoding module, and FSAS feature attention mechanism.

On the KWSD2 dataset, CM-UNetv2 surpasses all baseline methods in terms of mIoU and achieves a good balance between Precision and Recall. Although DeeplabV3+ slightly outperforms in precision, its mIoU and Recall are both lower than those of CM-UNetv2, reflecting its limited capability in handling elongated water bodies, occluded regions, or blurred boundaries. This performance gap is attributed to fundamental architectural differences: DeeplabV3+ relies on atrous convolutions to expand the receptive field but lacks an effective local detail aggregation mechanism. In contrast, CM-UNetv2’s DSSG module integrates two parallel feature streams through a dual-path connection, where one path is processed for semantic enhancement and the other retains the original encoder features. This approach improves the model’s ability to capture fine-grained structures and accurately represent details throughout the image.

Compared with Transformer-based models like SegFormer and TransUNet, CM-UNetv2 demonstrates more consistent and robust performance in Recall and mIoU metrics. While the Transformer architecture has strong long-range dependency modeling capabilities via global attention, it often struggles to capture local fine structures in images with blurred boundaries or dense small objects, leading to missed detections. For example, TransUNet performs relatively well on the MeiweiPCB industrial dataset but significantly declines on the KWSD2 dataset, indicating its structure is more suited for scenarios with clear boundaries and relatively concentrated target areas.

Both CM-UNet and CM-UNet++ adopt skip connection mechanisms, which offer notable advantages in terms of overall architecture. However, CM-UNetv2 further enhances the guidance capability of skip connection information by introducing the FGCMamba module in the decoder. This module leverages SGCA to efficiently extract spatial details, enabling the model to integrate both semantic information and fine-grained spatial features. It directs the most relevant encoder features to participate in detail reconstruction, achieving superior accuracy in boundary restoration and texture repair. Additionally, the FSAS module effectively integrates spatial and channel attention mechanisms, effectively highlighting key regions while suppressing redundant information in complex textures and background noise.

Furthermore, the performance differences across datasets reflect the coupling relationship between the network’s structural design and task attributes. For instance, TransUNet performs poorly in remote sensing tasks but ranks highly in industrial defect detection, indicating its structure is more suitable for close-range image processing with dense details and significant texture differences. CM-UNetv2, with its multi-scale fusion and global-local information collaboration mechanism, shows excellent adaptability in both task types.

Another notable advantage of the CM-UNetv2 architecture lies in its efficient embedding-depth configuration (embed dim = 96, depth = 2), which achieves the optimal balance on both datasets. This configuration ensures sufficient feature expression while maintaining manageable model complexity, enhancing training stability and inference efficiency.

In conclusion, CM-UNetv2 leads existing mainstream semantic segmentation methods in multiple key evaluation metrics. By implementing multi-scale skip information fusion via DSSG, improved contextual awareness via PPA, strengthening boundary guidance with FGCMamba, and focusing on key region feature expression using FSAS, it forms a high-performance semantic segmentation system that integrates global understanding and local modeling capabilities, demonstrating good scalability, generalization, and cross-domain adaptability.

## 6. Conclusions

We present CM-UNetv2, an advanced deep neural network for semantic segmentation designed to improve boundary delineation, regional consistency, and small-object detection. To overcome the limitations of traditional models in capturing fine-grained structures and contextual dependencies, CM-UNetv2 incorporates a set of dedicated modules, including the Patch-wise Patch Attention (PPA) for multi-scale feature extraction and the Dual-Stream Skip Guidance (DSSG) for semantic–spatial decoupling. In the decoding phase, the FGCMamba module integrates FSAS and SGCA to facilitate more effective feature fusion, enhancing contour accuracy and overall robustness.

Extensive experiments on the MeiweiPCB and KWSD2 datasets demonstrate that CM-UNetv2 achieves superior performance across key metrics, particularly in edge preservation and fine-structure recovery. However, we acknowledge certain limitations: the PPA module, while significantly boosting accuracy, introduces non-negligible computational overhead, which may impact real-time deployment in severely resource-constrained scenarios. Additionally, although CM-UNetv2 shows strong generalization on two datasets, its robustness across more diverse industrial defect types remains to be validated.

To address these limitations, future work will focus on: (1) designing a more lightweight version of PPA to improve runtime efficiency; (2) incorporating real-world PCB samples from industrial production lines to increase dataset diversity; and (3) extending the model into a multi-modal learning framework that integrates spectral, textural, or temporal cues. These enhancements aim to further improve the practicality and adaptability of our segmentation algorithm in complex industrial environments.

## Figures and Tables

**Table 1:** Results of CM-UNetv2 on the MeiweiPCB dataset and KWSD2 dataset.

Dataset	Precision	Recall	F1	mIOU
MeiweiPCB	0.8970	0.8882	0.8925	0.8224
KWSD2	0.9690	0.9822	0.9750	0.9516

**Table 2:** Results from the ablation study on network structure using the MeiweiPCB dataset.

Method	Precision	Recall	F1	mIOU
CM-UNet	0.8640	0.8580	0.8615	0.7932
CM-UNet+A	0.8680	0.8650	0.8645	0.8061
CM-UNet+B	0.8750	0.8755	0.8752	0.8099
CM-UNet+C	0.8685	0.8605	0.8620	0.7978
CM-UNet+A+B	0.8905	0.8800	0.8851	0.8125
CM-UNet+A+C	0.8845	0.8855	0.8850	0.8168
CM-UNet+A+B+C	0.8970	0.8882	0.8925	0.8224

**Table 3:** Results from the ablation study on network structure using the KWSD2 dataset.

Method	Precision	Recall	F1	mIOU
CM-UNet	0.9489	0.9412	0.9450	0.9205
CM-UNet+A	0.9540	0.9621	0.9580	0.9298
CM-UNet+B	0.9572	0.9604	0.9588	0.9336
CM-UNet+C	0.9510	0.9645	0.9577	0.9250
CM-UNet+A+B	0.9615	0.9695	0.9655	0.9388
CM-UNet+A+C	0.9624	0.9705	0.9665	0.9405
CM-UNet+A+B+C	0.9632	0.9720	0.9676	0.9516

**Table 4:** Results from the ablation study on Embedding–Depth Architecture using the MeiweiPCB dataset.

Embed Dim	Depths	Precision	Recall	F1	mIOU
4	64	0.8886	0.8840	0.8863	0.8178
3	128	0.8944	0.8850	0.8897	0.8199
2	96	0.8970	0.8882	0.8925	0.8224

**Table 5:** Results from the ablation study on Embedding–Depth Architecture using the KWSD2 dataset.

Embed Dim	Depths	Precision	Recall	F1	mIOU
4	64	0.9602	0.9690	0.9645	0.9495
3	128	0.9618	0.9705	0.9661	0.9505
2	96	0.9632	0.9720	0.9676	0.9516

**Table 6:** Comparative results with other semantic segmentation methods on the MeiweiPCB dataset.

Method	Precision	Recall	F1	mIOU
Deeplabv3+	0.8712	0.8576	0.8643	0.7855
SegFormer	0.8560	0.8415	0.8487	0.7730
TransUnet	0.8890	0.8785	0.8837	0.8115
Swinunet	0.8615	0.8470	0.8542	0.7783
CM-UNet	0.8640	0.8580	0.8615	0.7932
CM-UNet++	0.8732	0.8610	0.8670	0.8084
CM-UNetv2	0.8970	0.8882	0.8925	0.8224

**Table 7:** Comparative results with other semantic segmentation methods on the KWSD2 dataset.

Method	Precision	Recall	F1	mIOU
Deeplabv3+	0.9450	0.9410	0.9430	0.9320
SegFormer	0.9368	0.9491	0.9429	0.9158
TransUnet	0.9377	0.9432	0.9404	0.9223
Swinunet	0.9342	0.9481	0.9411	0.9186
CM-UNet	0.9489	0.9412	0.9450	0.9205
CM-UNet++	0.9549	0.9571	0.9560	0.9425
CM-UNetv2	0.9632	0.9720	0.9676	0.9516

**Table 8:** Computational complexity analysis measured on 256 × 256 inputs using a single NVIDIA 4090 GPU, with mIOU evaluated on the MeiweiPCB dataset.

Method	FLOPs (G)	Param. (M)	FPS	mIOU
Deeplabv3+	82.95	60.99	58.32	0.7855
SegFormer	17.84	47.22	43.31	0.7730
TransUnet	37.23	93.23	53.47	0.8115
Swinunet	46.52	149.10	31.29	0.7783
CM-UNet	13.28	13.11	60.74	0.7932
CM-UNet++	71.65	8.71	36.43	0.8084
CM-UNetv2	21.63	22.02	47.32	0.8224

**Table 9:** Complexity and inference speed of ablated CM-UNetv2 variants on MeiweiPCB (256 × 256, 4090 GPU).

Method	FLOPs (G)	Param. (M)	FPS
CM-UNetv2	21.63	22.02	47.32
w/o DSSG	20.54	21.28	50.12
w/o PPA	15.19	14.31	56.12
w/o FSAS	20.31	20.70	49.88

**Table 10:** Complexity and inference speed of ablated CM-UNetv2 variants on KWSD2 (256 × 256, 4090 GPU).

Method	FLOPs (G)	Param. (M)	FPS
CM-UNetv2	86.53	22.02	36.91
w/o DSSG	83.15	21.28	39.04
w/o PPA	57.97	14.31	44.27
w/o FSAS	81.25	20.70	38.73

## Data Availability

The raw data presented in this study will be available on request from the corresponding author.
